# Continuing the conversation about public health ethics: education for public health professionals in Europe

**DOI:** 10.1186/s40985-015-0001-4

**Published:** 2015-05-29

**Authors:** Lisa M Lee, Miguel Ángel Royo-Bordonada

**Affiliations:** 1 Executive Director, U.S. Presidential Commission for the Study of Bioethical Issues, Washington, DC USA; 2 Academic Director, National School of Public Health, Institute of Health Carlos III, Madrid, Spain

*We study virtues not to learn more about them, but to be more virtuous.* ---Aristotle In this issue of *Public Health Reviews*, we turn our attention again to public health ethics. In our 2012 issue dedicated to this topic, we published 17 papers on a variety of aspects of ethical public health practice. In that issue, Aceijas et al. surveyed European schools of public health and reported that of the 40 schools responding, a large majority stated that they included ethics in their academic programs [1]. However, the content and nature of teaching was variable and often taught in an unsystematic way. These results are similar to other studies conducted in the United Kingdom [2] and the United States [3]. European schools of public health expressed interest in improving the teaching of public health ethics and asked the Association of Schools of Public Health in the European Region (ASPHER) for support.

In response to this request, the editors of *Public Health Reviews* gathered papers that focus on teaching public health ethics. In the introductory piece, *Towards Public Health Ethics*, Royo-Bordonada and Roman-Maestre report on the relationship between practice and ethics, examining the raison d’être of ethics and providing a justification for the need for ethics in public health practice. This and the other papers included in this issue—developed in collaboration with the ASPHER Working Group on Ethics and Values in Public Health—examine various topics including what we should teach, how we should teach it.

An important related question is *why* we should teach public health ethics. Fundamentally, we must teach public health ethics because ethical practice creates and maintains public trust and public health cannot function without public trust. To serve the public—whether through controlling an outbreak of an infectious disease, preparing for or responding to public health emergencies, or reducing the impact of non-communicable diseases—communities and individuals must trust our decisions and actions. This trust grows in large part from past successes, transparent and participatory decision making, and ethical management of the inevitable moral tensions that arise in our work.

There is certainly no reason to believe that public health practitioners start from an unethical or immoral stance; quite the contrary, in fact, the vast majority of public health professionals are drawn to the field because of its noble mission. This mission includes a bent toward social justice, mitigating health disparities, and consideration of the community perspective. Solving challenging public health problems, however, requires more than a moral stance [4]. It often requires choosing between two undesirable actions or reconciling tension between competing reasonable interests. These types of ethical decision points are not exceptional; public health practitioners report facing such tensions and challenges in their daily work [5, 6]. Given the numerous and diverse stakeholders affected by public health interventions and the complex systems in which we work, public health professionals must have skills to negotiate solutions that meet diverse, and often conflicting needs. We must prepare public health professionals to move beyond a naïve approach of responding to ethical tensions according to their personal moral compass and equip them with skills to recognize ethical dimensions of their work, articulate them, consider possible paths forward, and implement decisions based on both scientific and ethical grounds [4].

We must, however, consider past ethical failures and abuses with which public health officials were directly or indirectly involved. While we believe that most public health professionals feel a calling to the mission of public health, the history of the field contains a number of serious ethical lapses. Some of these abuses were located in a particular institution or region and involved wrongful medical interventions—such as eugenics using sterilization and killing for “racial purification”—purportedly justified by a larger public health objective [7–9]. Some instances were more systemic abuses committed under the guise of “racial hygiene” with active and passive participation by the medical and public health communities. Most notably was the attempt to eliminate entire groups and categories of persons such as in the mass industrialized murder of Jews, gays, Poles, Gypsies, persons with physical and mental handicaps, and others during the Holocaust [7, 10, 11].

In the United States, the U.S. Public Health Service conducted a 40-year natural history study of syphilis between 1932 and 1972, withholding treatment from hundreds of African American men living in abject poverty in the rural South [12]. Also during that time, several of the same U.S. Public Health Service personnel conducted “ethically impossible” sexually transmitted infection studies in Guatemala, inoculating prisoners, commercial sex workers, psychiatric patients, and soldiers with syphilis, gonorrhea, and chancroid [13]. Similarly, ethically problematic research and attendant public discussion occurred in Europe at the turn of the 20^th^ century. A foundational case that prompted substantial improvements regarding consent for medical research featured Albert Neisser of the University of Breslau attempting to ‘vaccinate’ unsuspecting patients against syphilis using serum from infected persons [14]. Although protective requirements for participant consent resulted from the discovery of Neisser’s experiments, they were not different from consent requirements that were in place long before his research and the atrocities of World War II began. Whether these ethical failures were a result of a single bad actor or a set of systemic ills, they remind us that egregious ethical violations can and do occur [15].

The 2014 outbreak of Ebola virus in western Africa provides many reminders that policy makers and officials do not always carefully consider the ethical dimensions of the challenging decisions we must make in contemporary public health practice. There are clear humanitarian and global justice motivations to engage in the Ebola control efforts [16]. There have been some ethically questionable decisions, however, that illustrate the pressing need to consider and articulate the ethical dimensions of our work—attribution of blame onto infected health care workers, such as a nurse in Spain, [17] and ethically (and scientifically) questionable quarantine of health care workers returning home from affected regions, such as a nurse in the United States [18].

There is evidence that severe ethical errors start with something much smaller, much less harmful. A corner cut here, a shortcut there can lead, in rare circumstances, to dramatic misjudgment and harm [19]. Ensuring that future public health professionals are aware of past lapses in the field reminds us all of the present dangers of ethical hubris.

Public health ethics education is partly about understanding the ethical drivers of public health action, partly about professionalism—or demonstrating integrity as a public health practitioner, partly about research ethics, and partly about a process that helps us find a way forward amidst conflicting views about which option is best. Knowledge about and use of these tools are not optional, not if we are to gain and maintain the public trust that is essential for us to do our work. If having these skills is essential for us to do our work, then we must teach them.

Public health is a field that gains much of it strength from its multidisciplinary nature. It is this multidisciplinary perspective—the combination of health-related disciplines (medicine, veterinary medicine, and pharmacy, for example); social, behavioral, methodological, and laboratory sciences; and policy and law—that makes public health thrive [20]. Each of these disciplines is steeped in its own ethical traditions—some more explicit than others. As the field of public health brings these disciplines and their various ethical traditions together to solve population health challenges, it is critical to ensure that the synergies are maximized.

One important way to maximize these disciplinary synergies is to infuse ethics into public health practice, research, and policy from the start. This infusion will help dispel the myth that ethics is a tool to restrict, stop, or criticize public health activities or personnel. Early integration of an ethical perspective should facilitate, not obstruct our work. If we train public health professionals to anticipate ethical tensions, engage stakeholders, and deliberate possible solutions, we promote accountability and responsibility, and in turn gain public trust.

There are numerous approaches to teaching the many facets of public health ethics. One approach is to integrate ethical expectations both formally and through the hidden curriculum. Another is to combine an orienting course about various public health ethics topics with attention to ethical dimensions of each course or project. In this issue, Lindert et al. and Tulchinsky et al. describe the development of several contemporary approaches to teaching public health ethics. Potter describes a strategy and innovative didactic methods to inculcate ethical values effectively and efficiently, emphasizing the importance of repetition, teachers as role models, and courses which themselves demonstrate ethical practice. Finally, recognizing the development of a model public health ethics curriculum in the United States, Camps et al. make the case for a European public health ethics curriculum. They provide an array of recommendations, emphasizing the moral value that they see as best defining the European concept of public health as the common good, mutual aid, and a collective or shared responsibility for health.

Whatever the approach, it is imperative that public health educators across the globe actively teach public health ethics. Our desired outcome of ethically and scientifically competent graduates who can successfully negotiate an increasingly complex field depends on it.

*Note*: The views expressed here are those of the authors and do not necessarily represent the official position of the U.S. Presidential Commission for the Study of Bioethics Issues, the U.S. Department of Health and Human Services, or the Spanish National Health Service.
